# Strain-Specific Effects of *Bacillus velezensis* Cell-Free Supernatants on Canine Epithelial and Immune Cell Responses

**DOI:** 10.3390/microorganisms14071545

**Published:** 2026-07-15

**Authors:** Andreea Cornelia Udrea, Katrine Bie Larsen, Adrian Schwarzenberg, Steffen Yde Bak, Niels Christensen, Chong Shen

**Affiliations:** 1 Gut Immunology Laboratory, R&D, Health & Biosciences, International Flavors & Fragrance (IFF), Edwin Rahrs Vej 38, 8220 Brabrand, Denmark; andreea.cornelia.udrea@iff.com (A.C.U.); katrinebie.larsen@iff.com (K.B.L.); 2 Enabling Technologies, R&D, Health & Biosciences, International Flavors & Fragrance (IFF), Edwin Rahrs Vej 38, 8220 Brabrand, Denmarksteffen.yde.bak@iff.com (S.Y.B.); niels.christensen@iff.com (N.C.)

**Keywords:** *Bacillus velezensis*, strain specificity, canine proximal gastrointestinal epithelium, tight junctions, cytokine regulation

## Abstract

The probiotic potential of *Bacillus velezensis* is increasingly recognized in companion animal nutrition; however, the strain-specific mechanisms underlying host responses remain poorly understood. Here, we investigated metabolite-driven effects of three *B. velezensis* strains (LSSA01, 15AP4, and 2084) using cell-free supernatants (CFS) to decouple host responses from bacterial colonization. All findings are derived from in vitro models using cell-free supernatants (CFS), enabling assessment of metabolite-mediated effects under controlled conditions. Canine proximal epithelial (MCA-B1) cells and macrophage-like (DH82) cells were used to assess epithelial barrier regulation, cellular stress and apoptosis pathways, immune signaling, and innate effector function. LSSA01 CFS significantly enhanced transcription of epithelial junction and resilience markers, including claudin-1 (6.2-fold, *p* < 0.0001), ZO-1 (1.4-fold, *p* < 0.01), and BCL-2 (1.4-fold, *p* < 0.01), accompanied by an increased BCL-2/BAX ratio (*p* < 0.05), suggesting strengthened barrier function and enhanced expression of markers associated with cellular resilience. In contrast, 15AP4 and 2084 primarily modulated protein-level junctional organization and stress-response pathways and significantly enhanced macrophage phagocytic activity under inflammatory challenge (170–286% of control, *p* < 0.01–0.0001). Untargeted metabolomics revealed distinct strain-specific signatures, including marked enrichment of nucleotides such as adenosine monophosphate (31–39-fold vs. control, *p* < 0.0001), alongside vitamins and bioactive peptides. Together, these findings indicate that *B. velezensis* strains exhibit complementary, non-redundant metabolite-mediated activities on canine epithelial and immune cells, highlighting the importance of strain-resolved evaluation for mechanistic understanding and supporting future investigation, while acknowledging that in vivo validation will be important to confirm relevance for canine health applications.

## 1. Introduction

The gastrointestinal epithelium constitutes a dynamic interface between luminal microorganisms, dietary components, and the host immune system. In dogs, epithelial barrier integrity and tightly regulated immune signaling are central to gastrointestinal homeostasis, influencing nutrient absorption, microbial tolerance, and resilience to inflammatory or dietary stress [1,2]. Disruption of epithelial junctional organization, altered cytokine signaling, and dysregulated innate immune responses have been associated with acute diarrhea and chronic inflammatory enteropathies in canine populations, highlighting the importance of nutritional strategies that support epithelial stability and controlled immune activation [3,4]. Given the multifactorial nature of canine health, including interactions among host physiology, environmental influences, epithelial barrier function, and immune regulation, mechanistic studies using relevant canine models are essential for improving our understanding of factors that contribute to gastrointestinal homeostasis [5].

Spore-forming probiotics belonging to the genus *Bacillus* have attracted increasing attention in companion animal nutrition due to their environmental robustness and capacity to modulate host physiology beyond transient colonization. Unlike many non-sporulating probiotics, *Bacillus* species produce a wide range of extracellular enzymes, peptides, and low-molecular-weight metabolites during growth and sporulation, many of which interact directly with host epithelial and immune cells [6,7]. These secreted factors—often described as postbiotic components—have been shown in multiple mammalian cell systems to influence epithelial barrier function, redox homeostasis, and innate immune signaling independently of live bacterial contact [7,8].

Among *Bacillus* species, *Bacillus velezensis* has emerged as a probiotic of particular interest in canine gastrointestinal research. Controlled feeding trials and clinical investigations demonstrate that *B. velezensis* supplementation can support recovery from gastrointestinal disturbances, improve stool quality, and beneficially influence host–microbiota interactions in dogs [9,10]. Synbiotic approaches combining *B. velezensis* with dietary fibers have further been reported to improve immune-related biomarkers and maintain gut homeostasis under controlled feeding conditions [11]. Despite these favorable outcomes, *B. velezensis* is often evaluated as a single functional entity, without resolving the contribution of individual strains.

Increasing experimental evidence suggests that probiotic activity is strongly strain-dependent, even within a single species [6,7]. The three *B. velezensis* strains investigated in this study (LSSA01, 15AP4, and 2084) were selected to represent distinct biological entities within the same probiotic platform, differing in origin (proprietary vs. non-proprietary) and anticipated functional traits. Consistent with this, comparative genomic analyses indicate that *B. velezensis* strains share a conserved core genome but exhibit variability in accessory genes related to metabolism, stress response, and regulatory functions, which may contribute to functional divergence.

Although *B. velezensis* has been increasingly studied, strain-specific metabolite profiles and their functional relevance remain insufficiently characterized. Previous work, including swine studies [6], has demonstrated functional differences among strains in terms of host interaction and performance outcomes, suggesting that strain-dependent biological activity may be a consistent and reproducible phenomenon. However, the metabolite-mediated mechanisms underlying these differences remain unclear. Such variation may translate into divergent effects on epithelial tight-junction assembly, apoptosis–renewal balance, cytokine production, and immune regulation, and failure to resolve these differences may obscure mechanistic understanding and complicate rational probiotic formulation.

To specifically address this gap in metabolite-mediated mechanisms, cell-free supernatants (CFS) provide a controlled experimental platform independent of bacterial adherence, colonization, or proliferation. By isolating the secreted metabolic fraction, this approach enables direct assessment of postbiotic effects on host cells. Prior work in epithelial and immune co-culture models has shown that *B. velezensis*-derived CFS recapitulates many of the functional effects observed with live bacteria, underscoring secreted metabolites as a principal mediator of probiotic activity [6,7]. In the present study, we examined the strain-specific effects of CFS derived from three *Bacillus velezensis* strains—LSSA01, 15AP4, and 2084. Using canine epithelial (MCA-B1) and macrophage-like (DH82) cell models, we investigated how individual strains differentially modulate epithelial barrier components, apoptosis-associated pathways, cytokine responses, phagocytic activity, and metabolic profiles. By resolving functional outcomes at the strain level, this study aims to provide a clear understanding of strain-specific differences in *B. velezensis* and to support the evaluation of their distinct contributions to epithelial barrier function and immune responses in canine models.

## 2. Materials and Methods

### 2.1. Reagents and Materials

Unless otherwise specified, all cell culture reagents, plasticware, and laboratory consumables were obtained from Thermo Fisher Scientific (Roskilde, Denmark).

### 2.2. Cell Line and Culture Conditions

The canine proximal epithelial cell line MCA-B1 (ACC 828, DSMZ, Braunschweig, Germany) was propagated in DMEM/F12 medium supplemented with 10% heat-inactivated fetal bovine serum (FBS) and 1% penicillin–streptomycin (100 U/mL penicillin, 100 µg/mL streptomycin). The macrophage-like canine cell line DH82 (LGC Standards GmbH, Wesel, Germany; CRL-3590) was cultured under identical conditions. All cell cultures were maintained at 37 °C in a humidified atmosphere containing 5% CO_2_.

### 2.3. Probiotic Strains and Generation of Cell-Free Supernatants (CFS)

Three *Bacillus velezensis* strains—LSSA01, 15AP4, and 2084—were evaluated in this study. These strains are components of Enviva^®^ Pro (Danisco Animal Nutrition & Health, IFF, Oegstgeest, The Netherlands). LSSA01 and 15AP4 were isolated from turkey litter by Agtech Products (Manhattan, KS, USA) in 2002 and 2000, respectively. Strain 2084 is a commercially available, non-proprietary isolate sourced from Novonesis (Bagsværd, Denmark) and Microbial Discovery Group (Oak Creek, WI, USA).

Each strain was cultured aerobically overnight at 37 °C in Tryptic Soy Broth (TSB). Bacterial growth was monitored by measuring optical density at 600 nm (OD_600_) using an EnSight Multimode Plate Reader (PerkinElmer, Shelton, CT, USA). Cultures were centrifuged to remove cells, and the resulting supernatants were sterile-filtered through 0.2 µm vacuum filters (Thermo Scientific Nalgene aPES membrane, Thermo Scientific Nalgene, New York, NY, USA). Cell-free supernatants were prepared in batches, aliquoted, and stored until use; the same preparations were used across experiments to ensure consistency and minimize variability.

CFS was added to cell cultures at a maximum of 5% (*v*/*v*); prior testing confirmed that this did not alter the final medium pH, which remained between 7.0 and 7.2. Optical density values were correlated with colony-forming units (CFU) by serial dilution and plate counts (OD_600_ = 1.0 corresponding to approximately 1 × 10^9^ CFU/mL). Cultures were centrifuged and sterile-filtered to obtain cell-free supernatants (CFS). The resulting CFS preparations were diluted to a working concentration corresponding to the secretions derived from cultures standardized to 1 × 10^7^ CFU/mL prior to filtration. While this approach standardizes input across conditions, the composition and concentration of secreted metabolites may differ among strains, which were further explored through metabolomic analyses.

### 2.4. RT-qPCR Gene Expression Analysis

MCA-B1 cells were seeded in 96-well plates at a density of 2 × 10^5^ cells/mL and cultured at 37 °C until confluent. On the second day, cells were exposed to probiotic CFS corresponding to 1 × 10^7^ CFU/mL, as calculated from OD_600_ measurements. Control wells received DMEM containing TSB without CFS. Following overnight incubation in a 5% CO_2_ environment, cells were washed with phosphate-buffered saline (PBS) and lysed in 200 µL RLT buffer (Qiagen, Aarhus, Denmark) for 30 min at 37 °C. Proteinase K was subsequently added to a final concentration of 100 µg/mL.

RNA isolation and RT-qPCR analysis were conducted by Biotest (Trige, Denmark) using validated TaqMan assays (Supplementary Materials, Table S1), in accordance with previously established protocols [12]. Amplification efficiency and linearity were verified through standard curve analysis, with corresponding data provided in the Supplementary Information (Supplementary PCR STD Excel). Appropriate controls, including No Template Controls (NTC) and No Enzyme Controls (NEC), were included to monitor potential contamination and assess genomic DNA contribution, respectively. The stability of the selected housekeeping genes was confirmed prior to analysis to ensure reliable normalization. The gene expression panel included markers related to epithelial barrier integrity (CLDN1, OCLN, ZO-1, E-cadherin), apoptosis (BCL2, BAX), and inflammatory signaling (IL-1R1, IL-18). Nine biological replicates were analyzed per condition. Relative expression levels were normalized to three reference genes (Supplementary Materials, Table S1) using the formula below:

ΔCt = Ct_target − Ct_housekeeping.

Relative gene expression was calculated as Fold change = 2^−(ΔCt_sample−ΔCt_control)^ using the 2^−ΔΔCt^ method.

### 2.5. Proteomic Analysis of MCA-B1 Cells

MCA-B1 cells were seeded as described above and cultured to confluency. Media were refreshed, and CFS was added at a concentration equivalent to 1 × 10^7^ CFU/mL. Plates were incubated overnight at 37 °C, 5% CO_2_. Control wells contained DMEM + TSB only. Each condition included eight replicates. Cells were washed with PBS and lysed in a buffer containing 5% SDS, 100 mM triethylammonium bicarbonate, and protease inhibitors. Cell lysates were digested using a modified PAC protocol [13] adapted to a OT-2 robot (Opentrons Labworks, Inc., Long Island City, NY, USA). The total volume of cell lysates was reduced and alkylated using 5 mM TCEP and 10 mM CAA in 10 min at 90 °C. MagReSyn Amine (AMN005, LabLife Nordic AB, Danderyd, Sweden) beads of 15 µL were used for each digestion. We did overnight digestion at 37 °C. Peptides were transferred to a new plate and dried down in a speed vaccum. Peptide concentrations were estimated using a colorimetric BCA micro assay kit and samples adjusted to equal concentrations. A QC sample (mixture of all samples) was prepared by taking 10 µL from all samples. Peptides were analyzed using a Vanquish Neo UHPLC system (Thermo Scientific) coupled to a QExactive HF mass spectrometer (Thermo Fisher Scientific, Bremen, Germany). Samples were separated using a trap and elute configuration with a NanoViper trap column (Acclaim™ PepMap™ 100 C18, 3 µm particle size, i.d. 0.075 mm) and a Waters nanoEase M/Z Peptide BEH C18 column (Waters, Milford, MA, USA). A 70 min chromatographic gradient at 2000 nL/min was used for peptide separations. Mass spectrometer was operated in DDA mode with HCD fragmentation, selecting the top 12 most intense peptide ions per cycle. Samples were analyzed in a randomized series separated by a QC sample for every five samples. Raw data were processed in Proteome Discoverer v3.0 and searched against a UniProt *Canis familiaris* FASTA database containing 134,853 entries using an in-house Mascot server. Trypsin was chosen as the peptide with a maximum of 2 missed cleavages permitted. S-Carbamidomethyl cysteine was defined as a fixed modification and oxidation of methionine as a variable modification. The MS/MS results were searched with a peptide ion mass tolerance of ±10 ppm and a fragment ion mass tolerance of ±0.02 Da. Percolator was used for calculating false discovery rates (FDR) [14]. Only peptides identified as rank 1 peptides with a confidence value of 1% (q < 0.01) were considered for further analysis. Only proteins supported by rank 1 peptide identifications passing a 1% false discovery rate threshold (q < 0.01) were retained for downstream analyses. Label-free quantification (LFQ) was performed in Expressionist v.2025.1.3 (Genedata). Imported raw files were noise-filtered using a chemical noise subtraction. Chromatograms were retention-time aligned by a pairwise alignment, filtered and smoothed before peak detection, based on volumes. Detected peaks were isotopic-clustered and singletons were filtered out. Peak clusters were matched to the identifications from Proteome Discoverer. Proteins were quantified based on the three most intense peptides. Quantitative abundances were normalized by an intensity drift normalization, where intensities from QC samples were used to correct for drift in the nano LC-MS/MS analysis.

### 2.6. Untargeted Metabolomics of CFS

MCA-B1 cells were seeded in 96-well plates at 2 × 10^5^ cells/mL and grown to confluency. Fresh culture medium was applied, followed by the addition of CFS equivalent to 1 × 10^7^ CFU/mL. After 24 h of incubation at 37 °C, conditioned media were collected, vortex-mixed, centrifuged at 5000× *g* for 5 min, and filtered using a 96-well plate with 0.2 µm filters (Sigma, Kastrup, Denmark).

Samples were randomized and diluted 1:1 with H_2_O:MeOH using an Opentrons Flex liquid-handling platform (Opentrons Labworks, Inc., Long Island City, NY, USA). A pooled quality control (QC) sample was injected every 10 samples to monitor analytical performance and support batch correction, while blank samples were used to identify and remove background ions. Metabolite annotation was based on mass spectral library matching and corresponded to MSI level 2 identification.

Metabolomic profiling was performed using a 1290 Infinity III UHPLC (Agilent, Santa Clara, CA, USA) coupled to a timsTOF Flex MALDI 2 mass spectrometer (Bruker, Billerica, MA, USA). Separation was achieved on an Acquity UPLC HSS T3 column using a water–acetonitrile gradient containing 0.1% formic acid. The injection volume was 5 µL at a flow rate of 0.25 mL/min. Data were acquired in both positive and negative ionization modes using a VIP HESI source, with MS/MS spectra collected via PASEF across a mass range of 100–1350 m/z. Internal calibration employed sodium formate and an Agilent low-concentration tuning mix.

Raw data were processed in MetaboScape v2026 using the T-Rex 4D algorithm, which incorporates m/z, retention time, ion mobility, and signal intensity. Feature annotation leveraged combined in-house MS^2^ spectra and public databases, including GNPS, LipidBlast, Bruker NIST HRMS, MetaboBase Personal Library 3.0, and HMDB.

Metabolite annotations were assigned based on accurate mass, retention time, ion mobility characteristics, and MS/MS spectral matching against in-house and public databases where available. Metabolite identifications should therefore be considered putative unless confirmed using authentic reference standards.

Reported metabolite features passed the quality-control and filtering procedures implemented in MetaboScape prior to statistical analysis. PCA plots and heatmap were generated using MetaboAnalyst v4.0 (www.metaboanalyst.ca; accessed on 7 April 2026), data was normalized, Log transformed, and Pareto scaled.

### 2.7. Phagocytosis Assay

Phagocytosis by DH82 cells was evaluated using the Vybrant Phagocytosis Assay Kit (Thermo Fisher Scientific, Waltham, MA, USA). Cells were seeded at 2 × 10^5^ cells/mL in 96-well plates and incubated overnight. Media were replaced, and CFS was applied at a level corresponding to 1 × 10^7^ CFU/mL. LPS (50 ng/mL) was added concurrently. Cells were incubated for 24 h at 37 °C.

Fluorescein-labeled *E. coli* K-12 BioParticles were added and allowed to be phagocytosed for 2 h. Extracellular fluorescence was quenched with 0.4% trypan blue. Background fluorescence was determined using blank wells containing media only. The experiment was conducted in triplicate, yielding a total of 32 replicates. Fluorescence was measured at excitation/emission wavelengths of 480/520 nm, and phagocytic activity was calculated as:

Phagocytosis (%) = [(Fluorescence_test_ − Fluorescence_blank_)/(Fluorescence_untreated_ − Fluorescence_blank_)] × 100

### 2.8. Statistical Analysis

Both the metabolomics and proteomics data are analyzed using the multivariate method ANOVA Simultaneous Component Analysis (ASCA) allowing an overall multivariate significance test of control–treatment comparisons and for biomarker-variable selection. The false discovery rate (FDR) is latently controlled at 5%.

MetaboAnalyst v4.0 software (www.metaboanalyst.ca; accessed on 7 April 2026) is used for data analysis, and data was normalized, Log transformed, and Pareto scaled.

The remaining data are analyzed using the Kruskal–Wallis H test, a nonparametric one-way ANOVA on ranks, followed by Dunn’s multiple-comparison test for pairwise comparisons when a significant overall treatment effect was detected. The false discovery rate (FDR) was controlled at 5%, with a focus on control–treatment comparisons. GraphPad Prism v9 software was used for data analysis.

For all analyses, results were considered statistically significant at *p* < 0.05.

## 3. Results

### 3.1. Increase in MCA-B1 Expression of Tight Junction by B. velezensis Cell-Free Supernatant

Expression of epithelial tight and adherens junction-related genes (ZO-1, claudin-1, occludin, and E-cadherin) was quantified in MCA-B1 cells by RT-qPCR following exposure to cell-free supernatants from *Bacillus velezensis* strains LSSA01, 15AP4, and 2084. Gene expression values were normalized to the medium control, which was set to 1.0 ± SE.

**Figure 1:**
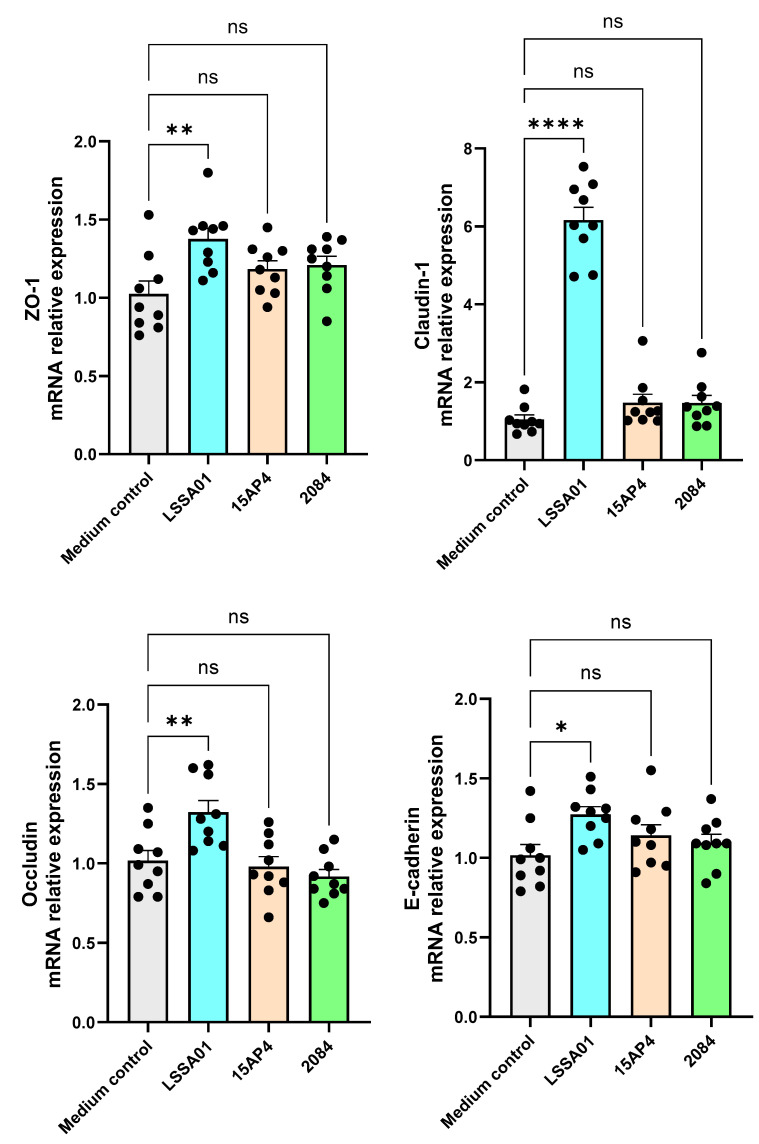
Effect of cell-free supernatant from *Bacillus velezensis* probiotics on barrier integrity in MCA-B1 cells, assessed by tight junction mRNA expression (ZO-1, CLDN-1, OCLN and E-cadherin), measured by RT-qPCR. Data are presented as fold changes relative to the medium control, with standard error (SE) bars. Each treatment group included nine biological replicates. Statistical significance is indicated as follows: ns, not significant; * *p* < 0.05; ** *p* < 0.01; **** *p* < 0.0001.

Treatment with LSSA01 resulted in significant upregulation of all four junction markers. ZO-1 expression increased to 1.4 ± 0.1 vs. medium control (1.0 ± 0.1; *p* < 0.01, Figure 1). A pronounced induction was observed for claudin-1, which reached 6.2 ± 0.3 vs. medium control (*p* < 0.0001). Occludin expression was also significantly elevated following LSSA01 treatment (1.3 ± 0.1 vs. medium control; *p* < 0.01), while E-cadherin showed a more moderate but still significant increase (1.3 ± 0.1 vs. medium control; *p* < 0.05).

In contrast, treatment with 15AP4 and 2084 resulted only in numerical changes for all four genes, and no statistically significant differences were detected (*p* > 0.05).

**Figure 2:**
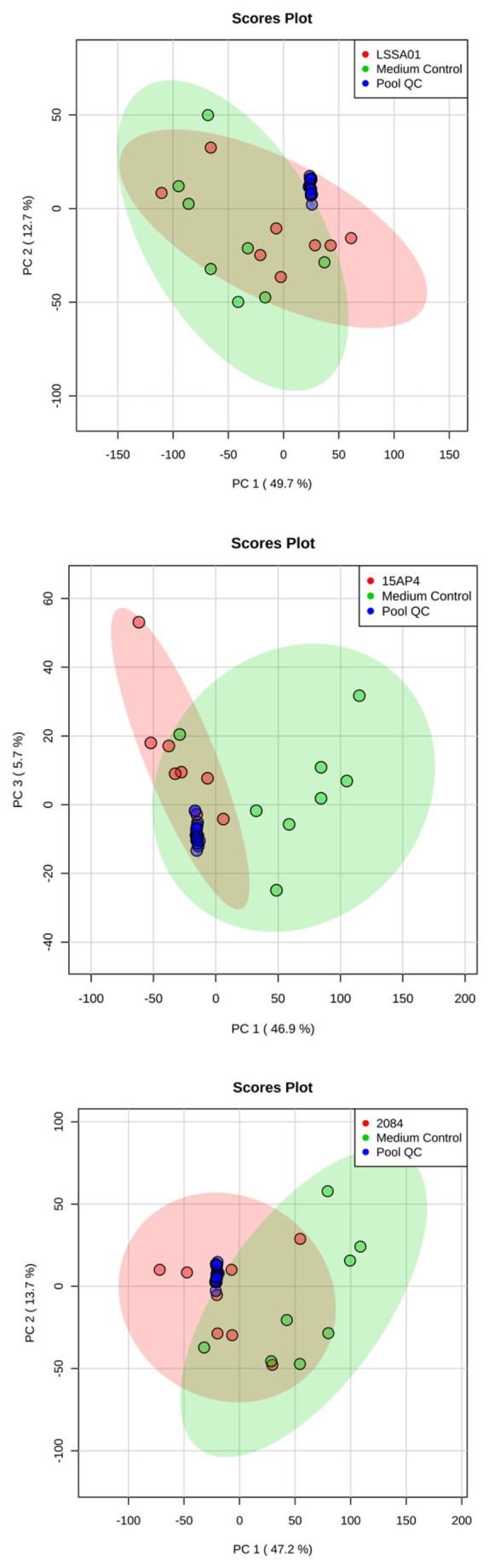
Principal component analysis (PCA) plots of proteomics data of MCA-B1 cells treated with cell-free supernatant (CFS) from three *Bacillus velezensis* strains, compared with medium control and Pooled QC samples. (**Upper**) Panel: LSSA01; (**Middle**) panel: 15AP4; (**Bottom**) panel: 2084.

Proteomic analysis was performed to quantify proteins involved in epithelial junction organization, including cadherin domain-containing protein, cingulin, gap junction protein, and tight junction protein 2, following exposure to cell-free supernatants derived from *Bacillus velezensis* strains LSSA01, 15AP4, and 2084. The PCA of the proteomics data shows how the data from the individual strains (Figure 2 first LSSA01, middle 2084 and bottom 15AP4) differs slightly in comparison to the medium control group. In all three plots, the pooled QC samples, which reflect the technical variation in the proteomic analysis, clustered closely together near the center of the plots, indicating good analytical reproducibility. For strains 2084 and 15AP4, a clear separation from the medium control was observed along PC1, which explained 47.2% and 46.9% of the total variance, respectively. To further evaluate the overall multivariate treatment effects, ANOVA Simultaneous Component Analysis (ASCA) was performed on the individual datasets [15]. The analysis showed no significant difference between LSSA01 and the medium control (*p* = 0.4), whereas significant differences were observed for both 2084 (*p* < 0.01) and 15AP4 (*p* < 0.001) compared with the medium control.

**Figure 3:**
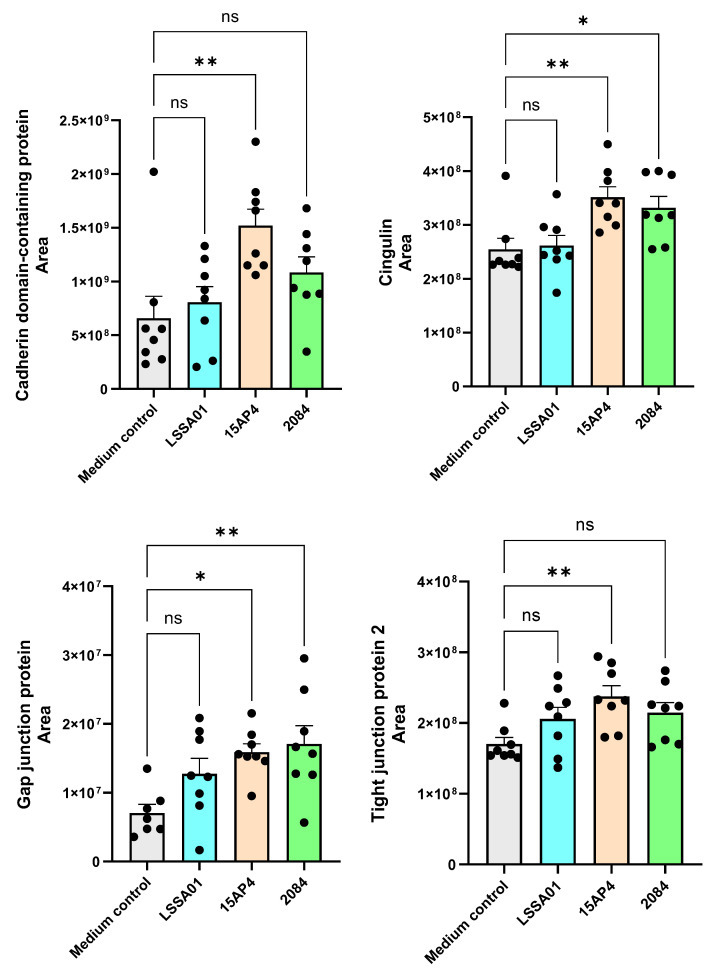
Protein abundance of cell junction markers (cadherin domain-containing protein, cingulin, gap junction protein and tight junction protein 2) in MCA-B1 cells. Values are means with associated standard error (SE) bars. The experiment was performed with 8 replicates per treatment group. ns, not significant; * *p* < 0.05; ** *p* < 0.01.

Protein abundance values were evaluated relative to the medium control and are summarized in Figure 3. For cadherin domain-containing protein, treatment with 15AP4 resulted in a significant increase in protein abundance compared with the medium control (1.3 ± 0.4 × 10^9^; *p* < 0.01), while LSSA01 and 2084 showed numerical but non-significant increases. Expression of cingulin was significantly elevated following treatment with 15AP4 (3.6 ± 0.5 × 10^8^; *p* < 0.01), double with a further significant increase observed after 2084 exposure (3.3 ± 0.4 × 10^8^; *p* < 0.05). No statistically significant difference was detected for LSSA01 relative to the medium control. For gap junction protein, both 15AP4 (1.6 ± 0.2 × 10^8^; *p* < 0.05) and 2084 (1.8 ± 0.2 × 10^8^; *p* < 0.01) induced significant increases in protein abundance compared with the medium control, whereas LSSA01 again showed only a numerical increase. In contrast, expression of tight junction protein 2 was significantly increased only following 2084 treatment (2.3 ± 0.1 × 10^8^; *p* < 0.01), with no statistically significant differences observed for LSSA01 or 15AP4 (Figure 3).

### 3.2. Modulation of Apoptosis-Related and Cellular-Resilience Markers in MCA-B1 Cells Treated with B. velezensis Cell-Free Supernatant

**Figure 4:**
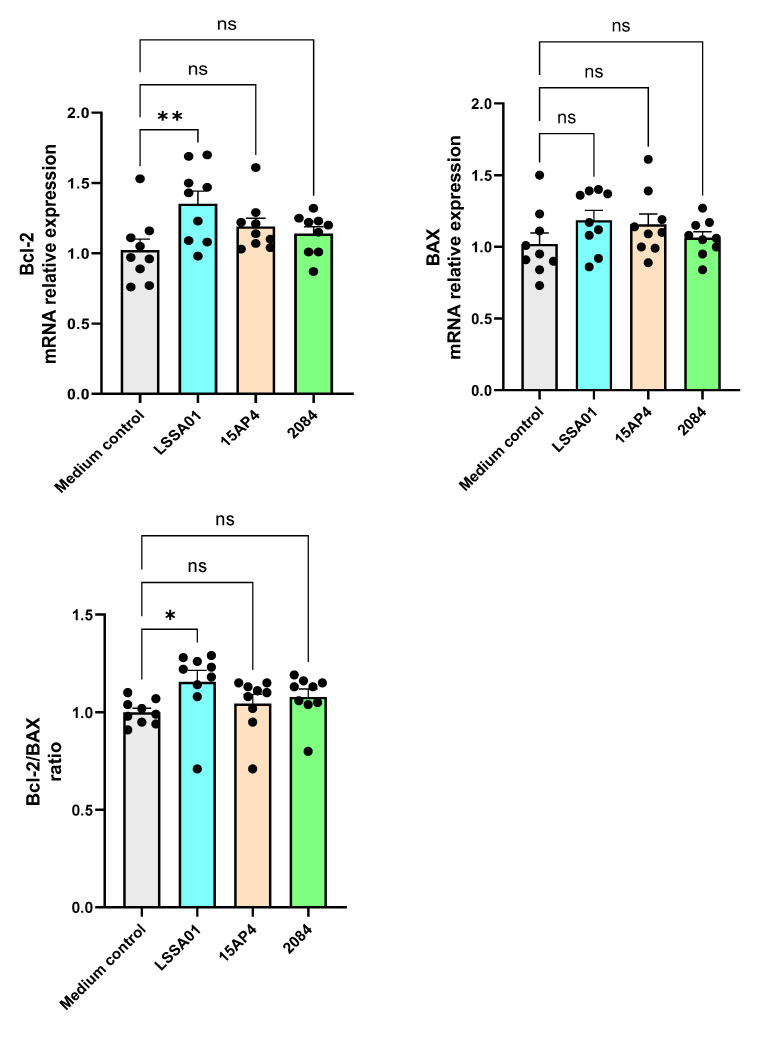
Effect of cell-free supernatant from *Bacillus velezenis* probiotics on mRNA expression of apoptosis-related genes (anti-apoptotic, Bcl-2; pro-apoptotic: BAX) and its ratio in MCA-B1 cells, measured by RT-qPCR. Data are presented as fold changes relative to the medium control, with standard error (SE) bars. Each treatment group included nine biological replicates. Statistical significance is indicated as follows: ns, not significant; * *p* < 0.05; ** *p* < 0.01.

The effects of *Bacillus velezensis* cell-free supernatant on apoptosis-related gene expression were evaluated in MCA-B1 cells by RT-qPCR, focusing on the anti-apoptotic marker Bcl-2, the pro-apoptotic marker BAX, and the Bcl-2/BAX ratio (Figure 4). Gene expression values were normalized to the medium control.

Expression of Bcl-2 was significantly increased following treatment with LSSA01 (1.4 ± 0.1 vs. medium control; *p* < 0.01), whereas treatment with 15AP4 and 2084 resulted in only numerical, non-significant changes compared with the control (Figure 4). In contrast, expression of the pro-apoptotic gene BAX did not differ significantly among treatment groups. Mean BAX expression levels remained close to those of the medium control following exposure to LSSA01, 15AP4, or 2084, with no statistically significant differences detected. Consistent with these findings, the Bcl-2/BAX ratio, an indicator of cellular susceptibility to apoptosis, was significantly increased only in cells treated with LSSA01 (1.2 ± 0.1 vs. medium control; *p* < 0.05). No significant changes in the Bcl-2/BAX ratio were observed following treatment with 15AP4 or 2084.

**Figure 5:**
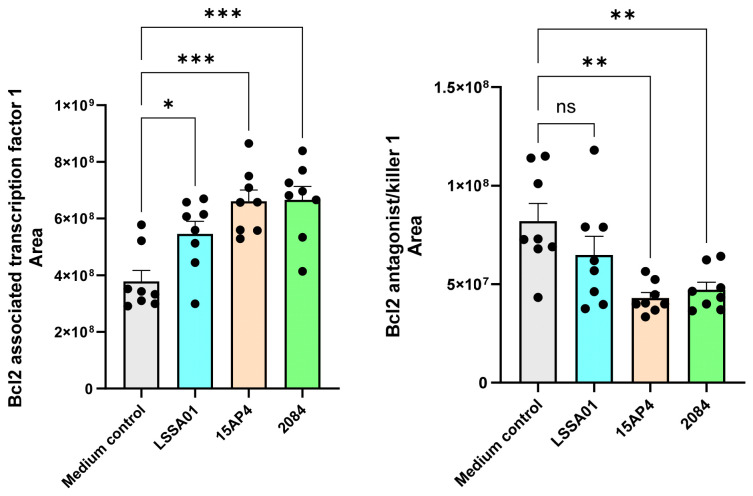

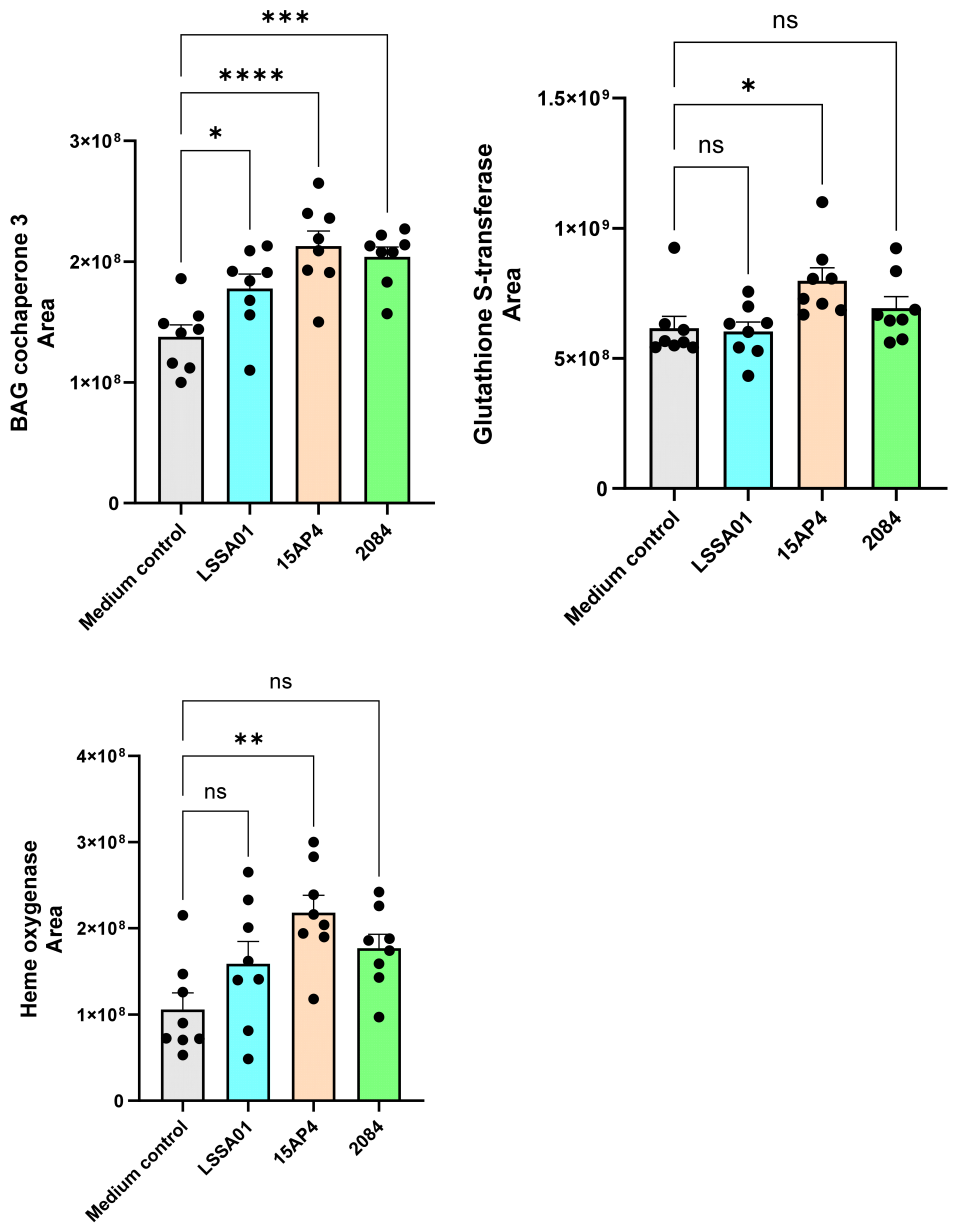
Protein abundance of anti-apoptosis (BCL2 associated transcription factor 1 and BCL2 antagonist/killer 1) and anti-stress markers (BAG cochaperone 3, Glutathione S-transferase and Heme oxygenase) in MCA-B1 cells. Values are means with associated standard error (SE) bars. The experiment was performed with 8 replicates per treatment group. ns, not significant; * *p* < 0.05; ** *p* < 0.01; *** *p* < 0.0001; **** *p* < 0.0001.

Proteomic analyses further corroborate the abundance of apoptosis- and stress-related proteins in MCA-B1 cells following treatment with *Bacillus velezensis* cell-free supernatants, including the anti-apoptotic regulators BCL2-associated transcription factor 1 and BCL2 antagonist/killer 1, as well as the anti-stress markers BAG cochaperone 3 (BAG3), a cytoprotective stress-response co-chaperone, glutathione S-transferase (GST), a detoxification enzyme, and heme oxygenase (HO-1), a stress-inducible cytoprotective enzyme (Figure 5).

Among the anti-apoptotic proteins, BCL2-associated transcription factor 1 abundance increased from 3.8 ± 0.4 × 10^8^ in the medium control to 5.5 ± 0.4 × 10^8^ with LSSA01 (*p* < 0.05), and further to 6.6 ± 0.4 × 10^8^ and 6.7 ± 0.5 × 10^8^ with 15AP4 and 2084, respectively (both *p* < 0.001; Figure 5). In contrast, levels of BCL2 antagonist/killer 1 decreased from 8.2 ± 0.9 × 10^7^ in the control group to 4.3 ± 0.3 × 10^7^ and 4.7 ± 0.4 × 10^7^ following treatment with 15AP4 and 2084, respectively (both *p* < 0.01), whereas no significant change was observed for LSSA01.

Consistent stress-response effects were observed for cytoprotective proteins. BAG3 abundance increased from 1.4 ± 0.1 × 10^8^ in the medium control to 1.8 ± 0.1 × 10^8^ with LSSA01 (*p* < 0.05) and showed a pronounced elevation to 2.1 ± 0.1 × 10^8^ and 2.0 ± 0.1 × 10^8^ following treatment with 15AP4 and 2084, respectively (*p* < 0.0001 and *p* < 0.001). For glutathione S-transferase, a significant increase was detected only after 15AP4 treatment (8.0 ± 0.5 × 10^8^ vs. 6.2 ± 0.5 × 10^8^ in controls; *p* < 0.05), while LSSA01 and 2084 did not differ significantly from the medium control. Similarly, heme oxygenase abundance increased significantly with 15AP4 (2.2 ± 0.2 × 10^8^ vs. 1.1 ± 0.2 × 10^8^; *p* < 0.01), with no statistically significant changes observed for LSSA01 or 2084.

### 3.3. Cell-Specific Immune Responses to Bacillus velezensis-Derived Metabolites: Cytokine Induction in MCA-B1 Cells and Phagocytosis in DH82 Cells

**Figure 6:**
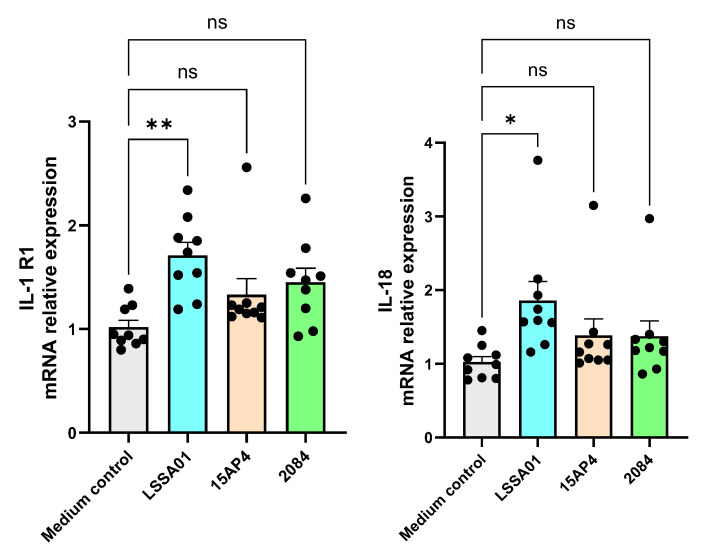
Effect of cell-free supernatant from *Bacillus velezensis* probiotics on the mRNA expression of immunomodulatory cytokines (IL-1R1 and IL-18) in MCA-B1 cells, measured by RT-qPCR. Data are expressed as fold change relative to the medium control, with standard error (SE) bars. Each treatment group included nine biological replicates. Statistical significance is indicated as follows: ns, not significant; * *p* < 0.05; ** *p* < 0.001.

To determine whether *Bacillus velezensis*-derived metabolites influence epithelial immune signaling, we examined the transcriptional response of MCA-B1 cells to probiotic cell-free supernatants, focusing on two cytokines central to cell-mediated immunity: IL-1 receptor type 1 (IL-1R1), a key mediator of IL-1–driven inflammatory signaling, and IL-18, an inflammasome-linked cytokine involved in innate immune activation (Figure 6). Transcript levels were quantified by RT-qPCR and normalized to the medium control. Expression of IL-1R1 was significantly increased in cells treated with LSSA01, reaching 1.7 ± 0.1 vs. 1.0 ± 0.1 in the medium control (*p* < 0.01; Figure 6). In contrast, treatment with 15AP4 (1.3 ± 0.2) and 2084 (1.5 ± 0.1) resulted in numerical increases that did not reach statistical significance compared with the control. A similar trend was observed for IL-18 expression. Transcript levels increased significantly following LSSA01 treatment (1.9 ± 0.3 vs. 1.0 ± 0.1 in the medium control; *p* < 0.05), whereas 15AP4 (1.4 ± 0.2) and 2084 (1.4 ± 0.2) did not induce statistically significant changes relative to the control.

**Figure 7:**
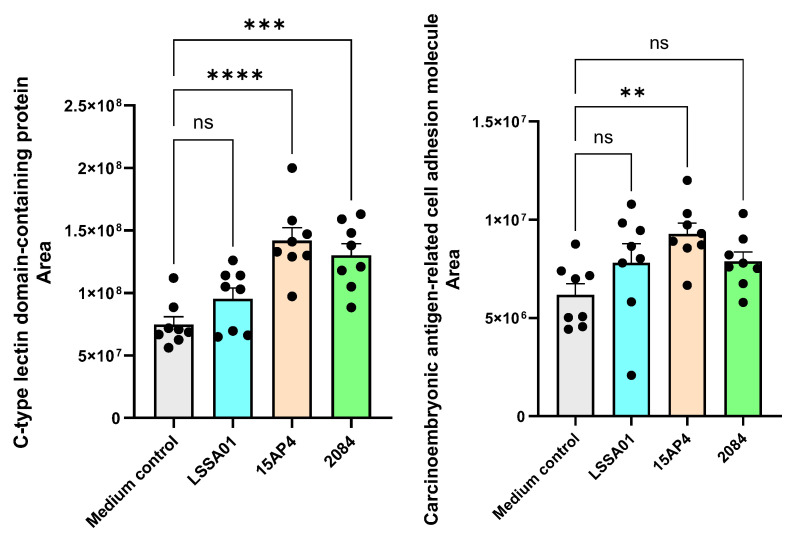
Proteomic abundance of immunostimulatory cytokine- and receptor-related proteins in MCA-B1 cells following treatment with cell-free supernatant from *Bacillus velezensis* probiotics. Proteins analyzed included C-type lectin domain-containing protein and carcinoembryonic antigen-related cell adhesion molecule (CEACAM). Data are presented as mean values with associated standard error (SE) bars. Each treatment group included eight biological replicates. Statistical significance is indicated as follows: ns, not significant; ** *p* < 0.01; *** *p* < 0.001; **** *p* < 0.0001.

To assess whether *Bacillus velezensis*-derived metabolites modulate epithelial immune recognition at the protein level, we quantified the abundance of two receptor-associated immunostimulatory proteins in MCA-B1 cells: C-type lectin domain-containing protein, a pattern-recognition receptor involved in microbial sensing, and carcinoembryonic antigen-related cell adhesion molecule (CEACAM), a mediator of immune-associated cell–cell interactions (Figure 7).

Protein abundance of the C-type lectin domain-containing protein increased in a strain-dependent manner. Mean abundance rose from 7.5 ± 0.6 × 10^7^ in the medium control to 9.5 ± 0.9 × 10^7^ following LSSA01 treatment. In contrast, significantly higher levels were observed after treatment with 15AP4 (1.4 ± 0.1 × 10^8^ vs. medium control; *p* < 0.0001) and 2084 (1.3 ± 0.1 × 10^8^ vs. medium control; *p* < 0.001). A more selective response was observed for CEACAM. Mean protein abundance increased from 6.2 ± 0.6 × 10^6^ in the medium control to 7.8 ± 1.0 × 10^6^ following LSSA01 treatment. A significant increase was detected only in cells treated with 15AP4, reaching 9.3 ± 0.5 × 10^6^ (*p* < 0.01 vs. medium control), whereas 2084 treatment (7.9 ± 0.5 × 10^6^) did not differ significantly from the medium control.

**Figure 8:**
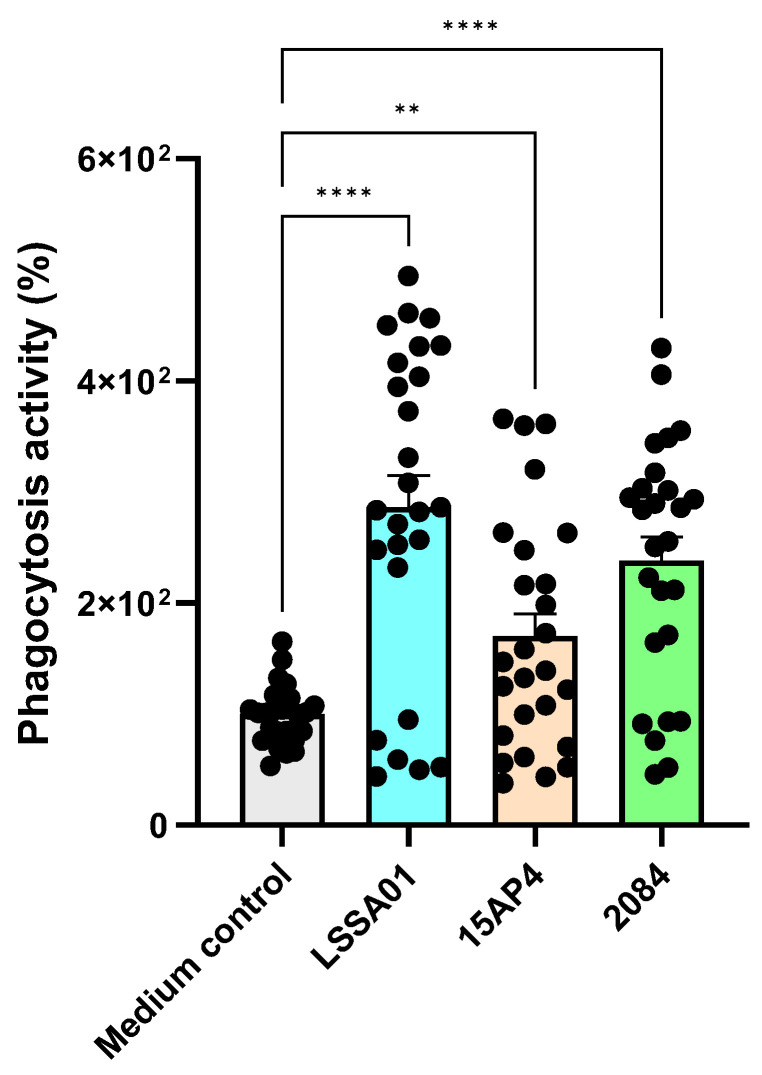
Phagocytic activity of LPS-stimulated DH82 macrophages following treatment with cell-free supernatants (CFS) derived from *Bacillus velezensis* probiotics. Phagocytic activity is expressed as percentage relative to the untreated medium control and presented as mean values with associated standard error (SE). The experiment was performed in three independent runs, yielding a total of 26 replicates per treatment group. Statistical significance is indicated as follows: ** *p* < 0.01; **** *p* < 0.0001.

To assess whether *Bacillus velezensis*-derived metabolites enhance innate immune effector function, the phagocytic activity of LPS-stimulated DH82 macrophages was measured following treatment with probiotic cell-free supernatants (Figure 8). Phagocytic activity is expressed as a percentage relative to the medium control.

Baseline phagocytic activity in the medium control was 100.0 ± 5.1%. Treatment with cell-free supernatant from LSSA01 resulted in a robust increase in phagocytosis, reaching 286.1 ± 28.6%, which was highly significant compared with the control (*p* < 0.0001). A significant enhancement in phagocytic activity was also observed following 15AP4 treatment, with mean values increasing to 170.0 ± 20.3% (*p* < 0.01). Similarly, exposure to 2084 markedly elevated phagocytic activity to 238.1 ± 21.6%, representing a highly significant increase relative to the medium control (*p* < 0.0001).

### 3.4. Immune-Regulatory Responses Induced by Bacillus velezensis-Derived Metabolites in MCA-B1 Cells

**Figure 9:**
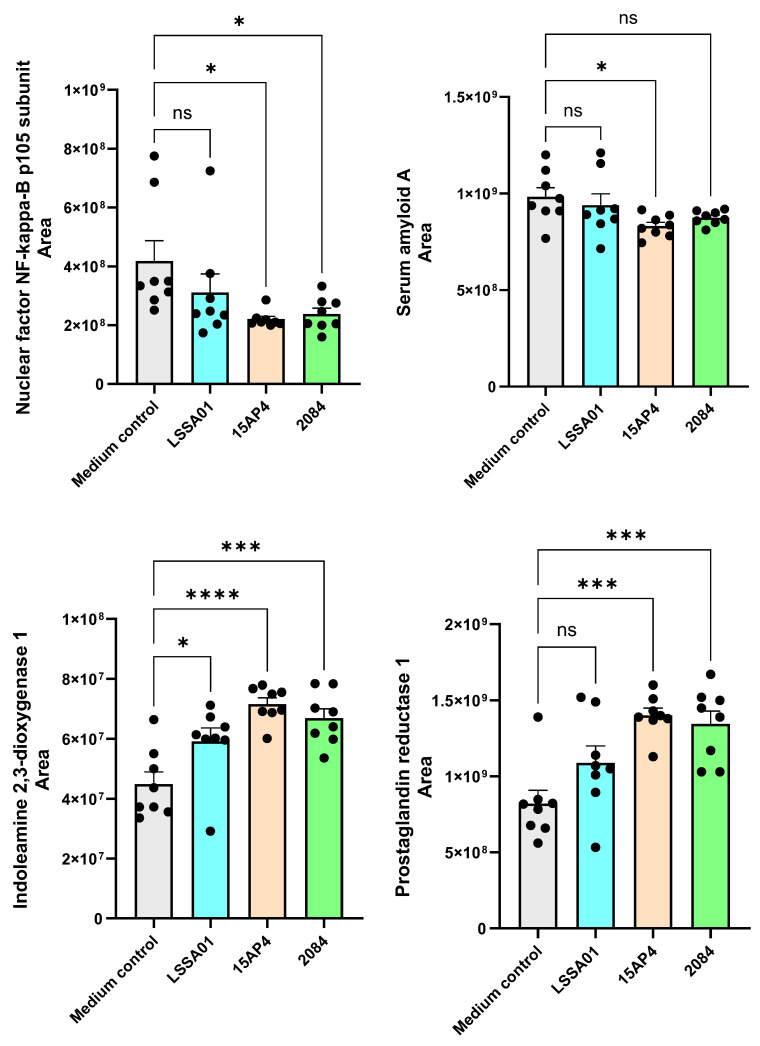
Proteomic abundance of immune-regulatory proteins in MCA-B1 cells following treatment with cell-free supernatants (CFS) derived from *Bacillus velezensis* probiotics. Proteins analyzed included NF-κB p105 subunit, serum amyloid A, indoleamine 2,3-dioxygenase 1, and prostaglandin reductase 1. Data are presented as mean values with associated standard error (SE) bars. Each treatment group included eight biological replicates. Statistical significance is indicated as follows: ns, not significant; * *p* < 0.05; *** *p* < 0.001; **** *p* < 0.0001.

To further evaluate whether *Bacillus velezensis*-derived metabolites modulate epithelial inflammatory regulation, we quantified the abundance of multiple immune-regulatory proteins in MCA-B1 cells following exposure to probiotic cell-free supernatants (Figure 9). This analysis included regulators of inflammatory transcription, acute-phase response proteins, and enzymes involved in immunoregulatory and lipid-mediated anti-inflammatory pathways.

Protein abundance of the NF-κB p105 subunit, a regulatory precursor that modulates NF-κB–dependent inflammatory signaling, was significantly reduced following treatment with 15AP4 (2.2 ± 0.1 × 10^8^ vs. 4.2 ± 0.7 × 10^8^ in the medium control; *p* < 0.05) and 2084 (2.4 ± 0.2 × 10^8^; *p* < 0.05), whereas LSSA01 did not differ significantly from the control (*p* = 0.3). A similar trend was observed for serum amyloid A (SAA), an acute-phase inflammatory protein. While 15AP4 significantly reduced SAA abundance (8.3 ± 0.2 × 10^8^ vs. 9.8 ± 0.5 × 10^8^; *p* < 0.05), no significant difference was detected following LSSA01 (*p* = 0.8) or 2084 (*p* = 0.2) treatment relative to the medium control. In contrast, proteins associated with immunoregulation and tolerance were upregulated. Abundance of indoleamine 2,3-dioxygenase 1 (IDO1), an enzyme involved in tryptophan metabolism and immune tolerance, increased significantly following LSSA01 (5.9 ± 0.5 × 10^7^; *p* < 0.05), with more pronounced increases observed for 15AP4 (7.2 ± 0.2 × 10^7^; *p* < 0.0001) and 2084 (6.7 ± 0.3 × 10^7^; *p* < 0.001) compared with the medium control. Likewise, prostaglandin reductase 1 (PTGR1), an enzyme involved in prostaglandin metabolism and regulation of inflammatory lipid mediators, was significantly increased following treatment with 15AP4 (1.4 ± 0.0 × 10^9^; *p* < 0.001) and 2084 (1.3 ± 0.1 × 10^9^; *p* < 0.001), while LSSA01 did not induce a statistically significant change relative to the medium control (*p* = 0.1).

### 3.5. Metabolomics Analysis in Bacillus velezensis and MCA-B1 Cell Coculture

**Figure 10:**
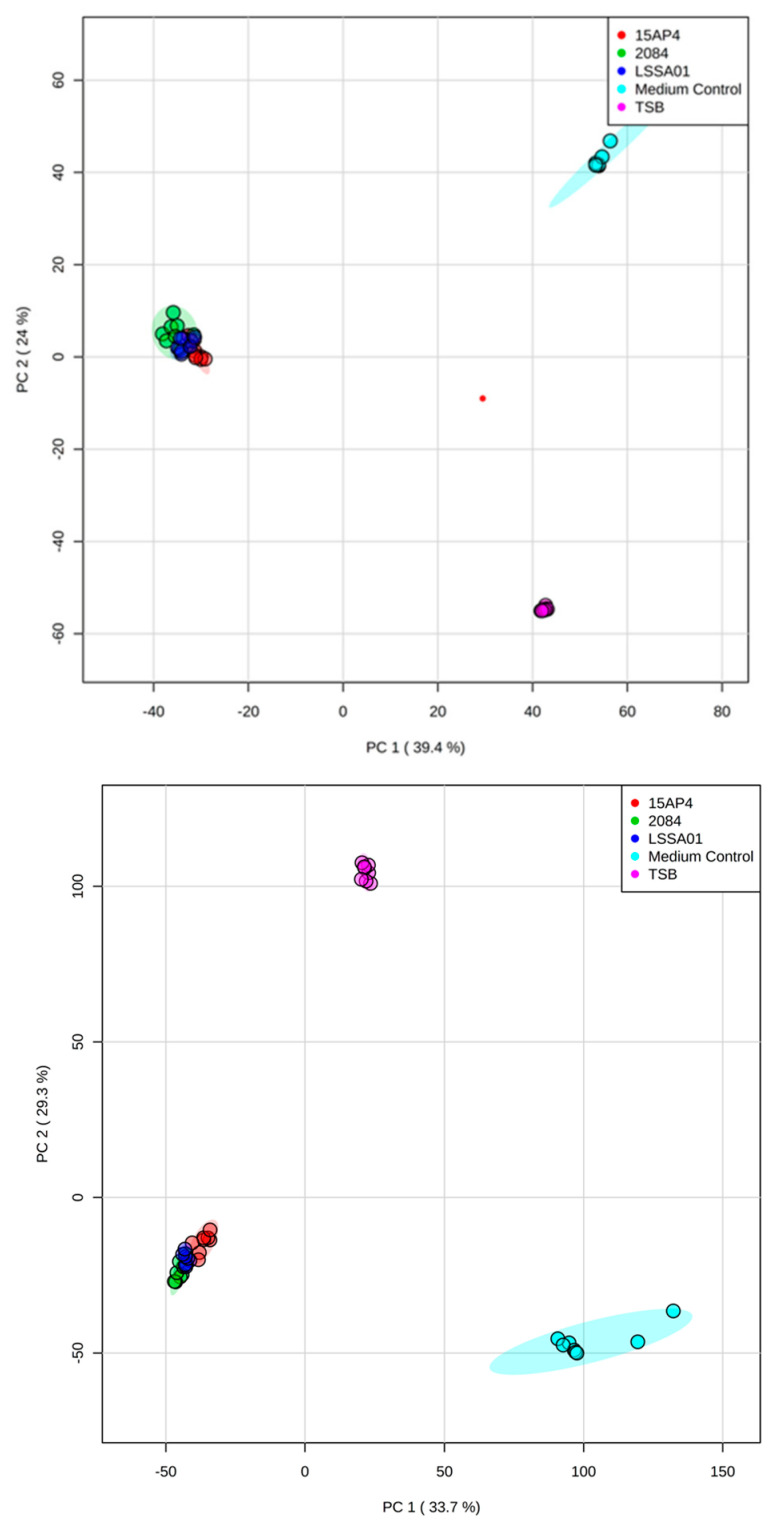
Principal component analysis (PCA) of untargeted metabolomics data from MCA B1 cells treated with cell free supernatant (CFS) from three *Bacillus velezensis* strains, compared with medium control and TSB, analyzed in positive (**upper**) and negative (**bottom**) ion modes.

Untargeted metabolomic analysis was performed to compare small-molecule metabolites produced by *Bacillus velezensis* strains LSSA01, 15AP4, and 2084 after 24 h incubation. The PCA analyses shown in Figure 10 summarize global metabolic changes in MCA B1 epithelial cells after treatment with a cell-free supernatant (CFS) from three *Bacillus velezensis* strains, compared with medium control and TSB (PC1 39.4% in positive mode and 33.7% in negative mode). In both ionization modes, samples cluster according to treatment, indicating that bacterial supernatants induce distinct metabolic responses compared with media controls, and that strain-specific effects are detectable.

**Figure 11:**
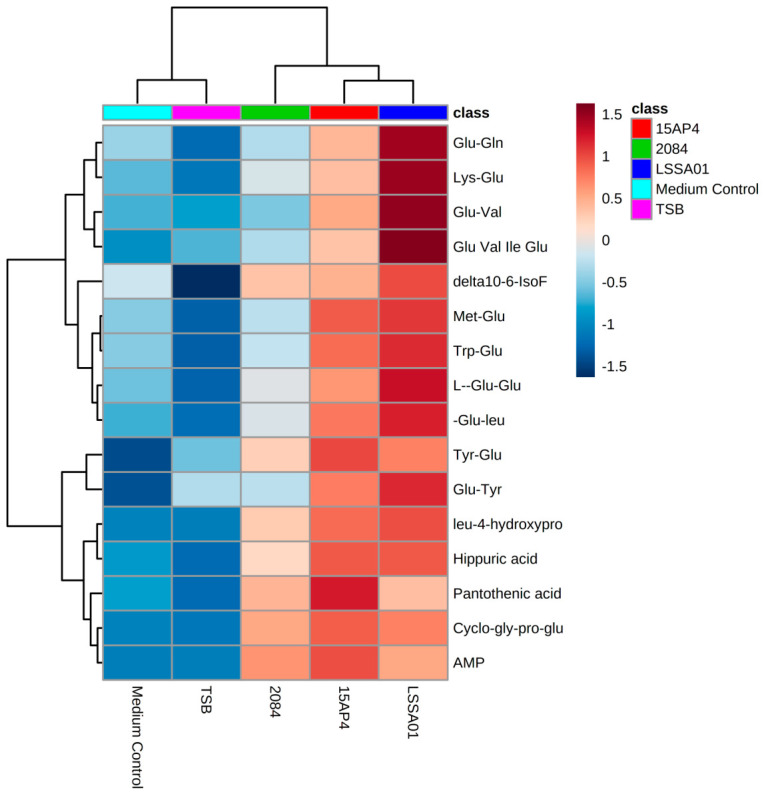
Heatmap of relative metabolite abundance across culture conditions. Heatmap showing relative abundance patterns of selected metabolites across medium control, TSB, and single-strain conditions (2084, 15AP4, and LSSA01). Metabolite intensities were averaged per condition and normalized.

Across the 16 identified metabolites, all three probiotic strains exhibited broad increases in metabolite abundance relative to the medium control, although the magnitude and significance of these changes varied by both metabolite class and strain (Figure 11). Several nucleotide- and vitamin-related metabolites showed particularly strong enrichment. For example, adenosine monophosphate (AMP) levels were markedly increased across all strains, reaching fold changes of approximately 31–39 relative to the control (mean metabolite abundances, fold changes relative to the medium control, and associated statistical significance are summarized in Supplementary Materials, Table S2), while pantothenic acid abundance increased between ~2- and 3-fold, with the highest levels observed in 15AP4.

Multiple dipeptides and amino acid-derived metabolites also suggested substantial strain-dependent enrichment. Leucyl-4-hydroxyproline, cyclo-(glycyl-prolyl-glutamyl), and Glu–Val–Ile–Glu exhibited large fold increases in all probiotic treatments, particularly in LSSA01 and 15AP4, with fold changes exceeding one order of magnitude relative to the medium control. Similarly, γ-glutamyl peptides (γ-Glu-Leu and L-γ-Glu-Glu) were consistently elevated across strains, indicating active peptide metabolism and secretion.

Differences between strains were also evident. 15AP4 often produced the highest absolute levels for several metabolites, including pantothenic acid and Glu-Tyr, while LSSA01 showed pronounced increases for selected oligopeptides such as Glu-Val–Ile–Glu. In contrast, metabolites such as Tyr-Glu showed minimal or non-significant changes relative to the control, highlighting metabolite-specific regulation rather than a uniform global increase.

## 4. Discussion

Dissecting the biological activity of probiotic formulations at the strain level is essential for understanding how microbial products influence host epithelial and immune function. While numerous studies describe beneficial gastrointestinal effects of *Bacillus*-based probiotics, most evaluate live bacteria or multi-strain formulations and therefore cannot resolve which functional attributes derive from individual strains or from emergent consortium behavior [7,16]. By focusing on cell-free supernatants, the present study isolates metabolite-driven host responses and provides insight into how distinct *Bacillus velezensis* strains engage epithelial barrier regulation, cellular stress responses, immune activation, and inflammatory modulation.

Strengthening of epithelial barrier integrity emerged as a unifying but strain-dependent outcome. LSSA01 acted predominantly at the transcriptional level, inducing robust expression of tight-junction and adherens-junction genes including CLDN1, OCLN, TJP1, and CDH1. This profile is consistent with reports in which *Bacillus velezensis* or *Bacillus subtilis* enhances epithelial junction gene expression through host energy-sensing pathways, particularly AMPK activation [17,18,19]. In contrast, strains 15AP4 and 2084 primarily elevated junction-associated proteins such as cingulin and TJP2 without parallel transcriptional shifts, suggesting post-transcriptional stabilization or enhanced junctional assembly. Similar dissociation between transcriptional and proteomic reinforcement has been observed in live *B. velezensis* ADS024 and in *Bacillus* consortia, where barrier integrity was preserved despite modest changes in epithelial mRNA abundance [7,16]. Importantly, the pronounced enrichment of AMP in LSSA01-derived supernatants may represent one potential contributor to the observed epithelial responses, as AMP-mediated AMPK activation has previously been associated with enhanced barrier function and epithelial recovery following stress [17,20]. Thus, our data suggest that LSSA01 exhibits a transcriptionally active profile associated with epithelial barrier support.

Differential modulation of apoptosis-related and stress-adaptive markers further distinguished strain efficacies. LSSA01 enhanced the expression of markers associated with cellular resilience, including increasing BCL2 expression and the BCL2/BAX ratio, a phenotype closely aligned with findings from ADS024 sterile filtrates, which suppress cleaved caspase-3 and apoptotic signaling in human colonic epithelia [12]. Conversely, 15AP4 and 2084 reduced abundance of pro-apoptotic regulators while substantially increasing cytoprotective proteins such as BAG3, glutathione S-transferase, and heme oxygenase. This strategy mirrors stress-buffering rather than anti-apoptotic priming and is consistent with studies showing that *Bacillus* metabolites enhance epithelial resilience through redox control and proteostasis rather than direct caspase inhibition [20,21]. The associated accumulation of γ-glutamyl peptides under 15AP4 and 2084 exposure is consistent with this interpretation, as these metabolites have previously been linked to glutathione metabolism and cellular redox regulation [21,22]. Their enrichment highlights a potentially relevant metabolic signature of these strains and warrants further investigation into their contribution to epithelial resilience and cellular stress responses.

Strain-dependent immune modulation further reinforced functional divergence. LSSA01 preferentially upregulated epithelial IL-1R1 and IL-18, suggesting enhanced innate sensing and epithelial immune readiness rather than overt immune activation. Controlled induction of IL-1 family signaling has been shown to promote epithelial vigilance while preserving barrier integrity, particularly under transient microbial exposure [23,24]. In contrast, 15AP4 and 2084 more potently enhanced immune execution and resolution, increasing abundance of pattern-recognition receptors and markedly boosting macrophage phagocytic activity under inflammatory challenge. These effects are concordant with in vivo and in vitro studies indicating that *Bacillus*-derived metabolites augment macrophage phagocytosis and bacterial clearance without exacerbating inflammation [25,26]. Simultaneous downregulation of NF-κB p105 and serum amyloid A, together with increased indoleamine-2,3-dioxygenase 1 and prostaglandin reductase 1, suggests modulation of pathways associated with immune regulation and maintenance of cellular homeostasis. These findings are consistent with previous observations that *Bacillus*-derived metabolites can influence host immune responses through multiple complementary mechanisms [3,4]. The enrichment of PEPT1-transportable oligopeptides and cyclic peptides may contribute to the observed immune responses; however, further studies are required to establish direct mechanistic links [27]. The strain-specific metabolite profiles identified in this study provide a valuable basis for generating mechanistic hypotheses and help guide future studies aimed at validating the contribution of individual metabolites to the observed cellular responses.

Taken together, these findings indicate that the three *B. velezensis* strains exhibit complementary, compensatory functional profiles rather than redundant activity. LSSA01 stands out for transcriptional barrier reinforcement and modulation of markers associated with cellular resilience, suggesting a potentially protective epithelial response profile under the conditions evaluated. In contrast, 15AP4 displays the broadest efficacy across stress tolerance, immune effector activation, and inflammatory resolution, while 2084 contributes similarly to immune modulation with comparatively lower epithelial transcriptional impact. This division of labor provides a mechanistic rationale for why *Bacillus* consortia often outperform single strains at the phenotypic level, while simultaneously highlighting the uncertainty inherent in predicting consortium performance from individual strain data alone. Indeed, recent in vitro comparisons of *B. velezensis* consortia demonstrate superior aggregate effects despite heterogeneity at the strain level, underscoring the emergent nature of multi-strain formulations [7].

Several limitations warrant consideration. Differences observed between transcriptional and proteomic readouts likely reflect the complementary nature of these analytical approaches, as gene expression and protein abundance are regulated at distinct biological levels and temporal scales [28]. While the measured transcripts and proteins represented related biological functions, they were not always identical targets. Therefore, complete concordance would not necessarily be expected. The observed differences among strains may indicate distinct yet complementary modes of action, with LSSA01 exhibiting stronger transcriptional responses and 15AP4 and 2084 eliciting more pronounced protein-level changes. Importantly, convergence was observed for key survival and stress pathways, including BCL-2-associated signaling and redox machinery, supporting the robustness of the findings. Nonetheless, the MCA-B1 and DH82 in vitro models do not capture the full complexity of the canine gastrointestinal environment, including mucus architecture, microbiota interactions, and immune cell crosstalk. Although these models provided a valuable and controlled platform for investigating strain-specific responses, future studies employing canine intestinal organoids, primary cells, and targeted pathway-validation approaches would further strengthen the physiological relevance of the findings and help clarify the contribution of host–microbiota interactions and specific signaling pathways. An additional consideration is that normalization of CFS based on CFU equivalents does not fully capture potential differences in metabolite composition or concentration across strains. These variations may contribute to the observed differences in host responses and reflect intrinsic strain-specific metabolic characteristics. Moreover, while cell-free supernatants enable precise attribution of host responses to secreted metabolites, they cannot address inter-strain metabolic interactions that may arise in consortia. Accordingly, in vivo validation—ideally integrating strain-resolved, consortia-level, and metabolomic analyses—will be essential to translate these mechanistic insights into predictive outcomes for canine health. Despite these limitations, the present study represents one of the most comprehensive strain-resolved, metabolites-focused analyses of *Bacillus velezensis* to date and provides a strong mechanistic foundation for rational strain selection and future in vivo evaluation.

## 5. Conclusions

This study indicates that *Bacillus velezensis* strains exhibit distinct, complementary postbiotic activities affecting epithelial barrier integrity, stress resilience, immune modulation, and metabolite production. These findings highlight the importance of strain-level evaluation and provide a biological framework for future mechanistic studies and strain-selection strategies.

## Data Availability

The datasets generated in this study are available upon request from the corresponding author.

## Supplementary Materials

The following supporting information can be downloaded at: https://www.mdpi.com/article/10.3390/microorganisms14071545/s1, Table S1: Taq Man ID numbers and housekeeping genes used in the RT-qPCR; Table S2: Differential metabolites produced by *Bacillus velezensis* strains after 24 h; Supplementary PCR STD Excel.

## Abbreviations

The following abbreviations are used in this manuscript:

AMP	Adenosine monophosphate
AMPK	AMP-activated protein kinase
BAK1	BCL-2 antagonist/killer 1
BCL2	B-cell lymphoma 2
CFS	Cell-free supernatant
CLDN1	Claudin-1
DH82	Canine macrophage-like cell line
IFNB1	Interferon beta-1
IL	Interleukin
IL-18	Interleukin-18
MCA-B1	Canine epithelial cell line
MyD88	Myeloid differentiation primary response 88
NF-κB	Nuclear factor kappa-B
PEPT1	Proton-coupled oligopeptide transporter 1
PTGR1	Prostaglandin reductase 1
qRT-PCR	Quantitative reverse-transcription PCR
STAT1	Signal transducer and activator of transcription 1
TJ	Tight junction
TSB	Tryptic soy broth
ZO-1	Zonula occludens-1
