# Supplementary material for: Strain-Specific Effects of Bacillus velezensis Cell-Free Supernatants on Canine Epithelial and Immune Cell Responses
Source: Microorganisms. 2026 Jul 15;14(7):1545. doi: 10.3390/microorganisms14071545 (PMC13414072; doi:10.3390/microorganisms14071545)
Supplement: Supplementary file 1 [file microorganisms-14-01545-s001.zip › microorganisms-4318859-supplementary.pdf]

Supplementary Materials

Table S1. Taq Man ID numbers and housekeeping genes used in the RT-qPCR.

Genome target in MCA-B1 cells	TaqMan ID No.
Claudin-1	Cf02713195_u1
Occludin	Cf02624089_m1
ZO-1	Cf01552709_m1
E-cadherin	Cf02697525_m1
IL-18	Cf02624262_m1
IL-1R	Cf02647245_m1
Bcl-2	Cf02622425_m1
BAX	Cf02727746_g1
**Housekeeping genes**	
ACTB	Cf04931159_m1
GAPDH	Cf04419463_gH
HPRT	Cf02690456_g1

Standard curve analysis of RT-qPCR assays used in this study. Amplification efficiency and linearity (R^2) were determined based on serial dilution of template material (Supplementary PCR STD Excel). All assays demonstrated acceptable performance within the experimental range.

Table S2. Differential metabolites produced by *Bacillus velezensis* strains after 24 h

Metabolite	TSB (mean ± SE)	Medium control (mean ± SE)	LSSA01			15AP4			2084		
			Mean ± SE	Fold	Statistics	Mean ± SE	Fold	Statistics	Mean ± SE	Fold	Statistics
Adenosine monophosphate	$2.1 \pm 0.1 \times 10^3$	$2.5 \pm 0.2 \times 10^2$	$7.6 \pm 0.0 \times 10^3$	30.6	$P < 0.001$	$9.7 \pm 0.1 \times 10^3$	39.0	$P < 0.001$	$8.0 \pm 0.0 \times 10^3$	32.4	$P < 0.001$
Pantothenic acid	$8.6 \pm 0.0 \times 10^4$	$2.7 \pm 0.0 \times 10^4$	$3.5 \pm 0.0 \times 10^5$	2.1	$P < 0.001$	$5.4 \pm 0.0 \times 10^5$	3.3	$P < 0.001$	$3.7 \pm 0.0 \times 10^5$	2.2	$P < 0.05$
Glu–Gln	$3.4 \pm 0.0 \times 10^4$	$3.4 \pm 0.0 \times 10^4$	$5.3 \pm 0.0 \times 10^4$	1.6	$P < 0.001$	$4.6 \pm 0.0 \times 10^4$	1.3	$P < 0.01$	$4.1 \pm 0.0 \times 10^4$	1.2	$P < 0.05$
L-γ-Glu-Glu	$1.0 \pm 0.0 \times 10^4$	$5.2 \pm 0.0 \times 10^3$	$2.2 \pm 0.0 \times 10^4$	4.3	$P < 0.001$	$1.9 \pm 0.0 \times 10^4$	3.7	$P < 0.001$	$1.6 \pm 0.0 \times 10^4$	3.1	$P < 0.001$
Tyr-Glu	$4.6 \pm 0.0 \times 10^4$	$4.9 \pm 0.0 \times 10^4$	$4.5 \pm 0.0 \times 10^4$	0.9	ns	$4.3 \pm 0.0 \times 10^4$	0.9	ns	$3.8 \pm 0.0 \times 10^4$	0.8	$P < 0.05$
Glu-Tyr	$8.0 \pm 0.1 \times 10^4$	$1.9 \pm 0.1 \times 10^4$	$1.3 \pm 0.2 \times 10^5$	6.8	$P < 0.001$	$1.1 \pm 0.2 \times 10^5$	6.1	$P < 0.001$	$8.1 \pm 0.2 \times 10^4$	4.3	$P < 0.01$
Lys-Glu	$8.7 \pm 0.2 \times 10^2$	$8.2 \pm 0.2 \times 10^2$	$3.5 \pm 0.0 \times 10^3$	4.3	$P < 0.001$	$2.1 \pm 0.0 \times 10^3$	2.6	$P < 0.001$	$1.5 \pm 0.0 \times 10^3$	1.9	$P < 0.05$
Leucyl-4-hydroxyproline	$1.3 \pm 0.0 \times 10^3$	$1.1 \pm 0.0 \times 10^3$	$2.0 \pm 0.0 \times 10^4$	18.6	$P < 0.001$	$1.9 \pm 0.0 \times 10^4$	17.4	$P < 0.001$	$1.4 \pm 0.0 \times 10^4$	12.6	$P < 0.001$
Hippuric acid	$2.4 \pm 0.0 \times 10^3$	$1.2 \pm 0.0 \times 10^3$	$1.2 \pm 0.1 \times 10^4$	9.6	$P < 0.01$	$1.2 \pm 0.1 \times 10^4$	9.6	$P < 0.01$	$7.8 \pm 0.1 \times 10^3$	6.5	$P < 0.05$
Δ^{10}-6-IsoF	$1.8 \pm 0.0 \times 10^3$	0	$3.0 \pm 0.0 \times 10^3$	—	$P < 0.001$	$2.5 \pm 0.0 \times 10^3$	—	$P < 0.001$	$2.3 \pm 0.0 \times 10^3$	—	$P < 0.001$

Glu-Val	$4.3 \pm 0.1 \times 10^4$	$2.2 \pm 0.1 \times 10^3$	$4.1 \pm 0.1 \times 10^4$	18.2	$P < 0.001$	$2.3 \pm 0.1 \times 10^4$	10.4	$P < 0.01$	$5.6 \pm 0.1 \times 10^3$	2.5	ns
Met-Glu	$1.9 \pm 0.1 \times 10^4$	$2.2 \pm 0.1 \times 10^3$	$5.2 \pm 0.1 \times 10^4$	23.8	$P < 0.001$	$4.8 \pm 0.1 \times 10^4$	22.2	$P < 0.001$	$2.3 \pm 0.1 \times 10^4$	10.9	$P < 0.01$
Cyclo-(Gly-Pro-Glu)	$2.5 \pm 0.0 \times 10^3$	$7.7 \pm 0.0 \times 10^2$	$3.5 \pm 0.0 \times 10^4$	13.6	$P < 0.001$	$3.8 \pm 0.0 \times 10^4$	14.8	$P < 0.001$	$3.1 \pm 0.0 \times 10^4$	12.1	$P < 0.001$
Glu-Val-Ile-Glu	$1.8 \pm 0.1 \times 10^4$	$4.9 \pm 0.0 \times 10^2$	$1.5 \pm 0.2 \times 10^5$	299	$P < 0.001$	$7.6 \pm 0.1 \times 10^4$	154	$P < 0.001$	$3.9 \pm 0.1 \times 10^4$	80	$P < 0.001$
γ-Glu-Leu	$1.3 \pm 0.0 \times 10^4$	$4.7 \pm 0.0 \times 10^3$	$7.7 \pm 0.1 \times 10^4$	6.0	$P < 0.001$	$6.4 \pm 0.1 \times 10^4$	5.0	$P < 0.001$	$3.8 \pm 0.1 \times 10^4$	2.9	$P < 0.05$
Trp-Glu	$3.0 \pm 0.0 \times 10^3$	$7.0 \pm 0.0 \times 10^2$	$8.3 \pm 0.0 \times 10^3$	11.8	$P < 0.001$	$7.2 \pm 0.0 \times 10^3$	10.3	$P < 0.001$	$3.8 \pm 0.0 \times 10^3$	5.4	$P < 0.01$

Values are presented as mean ± SE ($n = 8$). Fold change and P-values were calculated relative to the medium control. ns, not significant.
